# Descriptive Epidemiology of and Response to the High Pathogenicity Avian Influenza (H5N8) Epidemic in South African Coastal Seabirds, 2018

**DOI:** 10.1155/2023/2708458

**Published:** 2023-02-23

**Authors:** Laura C. Roberts, Celia Abolnik, Lauren J. Waller, Kevin Shaw, Katrin Ludynia, David G. Roberts, Alison A. Kock, Azwianewi B. Makhado, Albert Snyman, Darrell Abernethy

**Affiliations:** ^1^Department of Production Animal Studies, Faculty of Veterinary Science, University of Pretoria, Pretoria 0110, South Africa; ^2^Department of Agriculture, Western Cape Government, Elsenburg 7607, South Africa; ^3^CapeNature, Cape Town 7766, South Africa; ^4^Department of Biodiversity & Conservation Biology, University of the Western Cape, Bellville 7535, South Africa; ^5^Southern African Foundation for the Conservation of Coastal Birds (SANCCOB), Cape Town 7441, South Africa; ^6^Department of Biological Sciences, University of Cape Town, Rondebosch 7701, South Africa; ^7^Cape Research Centre, South African National Parks, Tokai 7966, South Africa; ^8^South African Institute for Aquatic Biodiversity, Grahamstown 6140, South Africa; ^9^Republic of South Africa Department of Forestry, Fisheries and the Environment, Cape Town 8001, South Africa; ^10^FitzPatrick Institute of African Ornithology, University of Cape Town, Rondebosch 7701, South Africa; ^11^Aberystwyth School of Veterinary Science, Institute of Biological, Environmental and Rural Sciences, Aberystwyth University, Aberystwyth SY23 3DA, UK

## Abstract

High pathogenicity avian influenza (HPAI) clade 2.3.4.4b H5N8 virus was detected in coastal seabirds in late 2017 in South Africa, following a devastating epidemic in the commercial poultry and ostrich industries. By May 2018, the infection had been confirmed in fifteen seabird species at 31 sites along the southern coast, with the highest mortality recorded in terns (Family *Laridae*, Order *Charadriiformes*). Over 7,500 positive or suspected cases in seabirds were reported. Among those infected were three endangered species: African penguins (*Spheniscus demersus* Linnaeus, 1758), Cape cormorants (*Phalacrocorax capensis* Wahlberg, 1855), and Cape gannets (*Morus capensis* Lichtenstein, 1823). The scale and impact of this outbreak were unprecedented in southern African coastal seabirds and raised logistical challenges in resource allocation, risk mitigation, and outbreak response. It required the collaboration of multiple stakeholder groups, including a variety of government departments and nongovernmental organizations. With another HPAI outbreak in South African seabirds in 2021 and major incursions in seabird species in the northern hemisphere in 2022, it is vital to share and consolidate knowledge on the subject. We describe the epidemic, the lessons learned, and recommendations for developing contingency plans.

## 1. Introduction

Avian influenza (AI) is a viral disease of birds transmitted through faeces and respiratory secretions. High pathogenicity avian influenza (HPAI) strains cause severe illness and high mortality in poultry but have variable effects in other avian species, from no clinical signs to high mortality rates [1]. The management of HPAI outbreaks in poultry is subject to international and national government regulations and includes quarantining of infected farms, culling of poultry, and vaccination in some countries.

From approximately 2016, avian influenza Goose/Guangdong (Gs/GD)-lineage clade 2.3.4.4b H5N8 viruses caused the fourth and, until then, most severe global HPAI wave in terms of the numbers of wild birds and poultry farms affected [2]. The first detection was in wild water birds in May 2016 in Russia and China, and the virus subsequently spread across Asia and Europe and into Africa over the following year. The deaths of 60% of 2000 white-winged black terns (*Chlidonias leucopterus* Temminck, 1815) in early January 2017 on Lake Victoria in Uganda were attributed to H5N8 HPAI [3–5]. In June 2017, HPAI (H5N8) reached South Africa, causing widespread outbreaks in commercial poultry, backyard/hobby birds, and wild birds until October 2017 [5]. Five distinct genetic variants of the virus were detected in the northern half of South Africa in 2017, but only one of these reached the southern provinces [6].

Africa acts as an ecological sink for Gs/GDH5Nx HPAI viruses spread by wild birds from North-Central Asia, the Middle East, and Europe. West Africa, with its extensive permanent wetlands, acts as the central hotspot for virus introduction and dissemination in the continent, and genetic data indicate that the 2017 H5N8 HPAI outbreaks in South Africa were most likely introduced from West Africa with intra-African migrant ducks [7, 8]. Locally, ubiquitous bridge species such as sacred ibis (*Threskiornis aethiopicus* Latham, 1790) and Egyptian geese (*Alopochen aegypticus* Linnaeus, 1766) could have introduced the virus to coastal species. Phylogenetic and time to the most recent common ancestor analyses on coastal bird viral genomes have revealed that the HPAI virus affecting coastal birds most likely emerged in October 2017 [9].

This paper describes the distribution of H5N8 HPAI in coastal seabirds in South Africa in 2018, the response to the outbreak, and the lessons learned. Devastating outbreaks in seabirds in South Africa in 2021 and in the northern hemisphere in 2022 [10–12] have highlighted the need to share and consolidate knowledge about preparedness and response measures required to manage such events. Though general guidelines for outbreak response are available [13–15], published accounts of practical experience are rare [16].

## 2. Material and Methods

A suspected case was defined as an individual bird with clinical signs indicative of avian influenza or found as a carcass, with no other apparent cause of death, between 1 December 2017 and 31 May 2018. Initial suspected cases from new species and sites were prioritised for sampling and testing to save limited government resources. However, anyone could submit samples and pay for testing, so sampling was not always restricted in this way.

A confirmed or “positive” case was defined as an individual bird from which an organ sample, brain, cloacal or tracheal/oropharyngeal swab sample, or any pooled samples tested positive on influenza A virus real-time reverse transcription polymerase chain reaction (rRT-PCR), and H5-orN8-specific rRT-PCR. The influenza A rRT-PCR was performed using the VetMAX™Gold AIV detection kit (Thermo Fisher: Life Technologies, Cat. no. 4485261) according to the manufacturer's protocol. The kit targets the matrix protein 1, matrix protein 2, and nucleoprotein genes of influenza A viruses. Cycle threshold (Ct) values < 36 were considered positive. The VetMax-Plus One-Step RT-PCR Kit (Cat. No. 4415328) was used for the H5 and N8 tests. Avian influenza virus (AIV) H5 subtype detection was carried out using the primers and probes described by Slomka et al. [17, 18], and Ct ≤ 35 was considered positive. AIV N8 detection was investigated using primers and probes described by Hoffman et al. [19, 20], considering Ct ≤ 36 as positive. Suspected cases that then tested negative were no longer counted as suspected.

Samples were collected by state veterinarians at the Western Cape Provincial Veterinary Laboratory (WCPVL), veterinary or research staff at rehabilitation centres, and occasionally by private vets and field staff. Samples and carcasses were submitted to the WCPVL, or in 12% of positive cases, to a private laboratory; Deltamune, Oudtshoorn. Brain samples were obtained by making a small incision in the skin and skull on the top of the head and inserting and agitating a cotton swab with a plastic handle to collect brain tissue.

Identification of suspected cases relied on reports submitted by the public when they happened to observe suspicious birds or by the staff of the managing conservation authorities. These staff reported observations made either during scheduled patrols or during other daily activities. Daily monitoring took place at one of the two mainland penguin colonies and on three manned islands but was carried out from a distance where species such as cormorants had dense breeding colonies. Other sites had at least weekly patrols that were, in some cases, performed more frequently during the outbreak.

A reporting form for gathering morbidity and mortality data was prepared by provincial veterinary services and provided to managing authorities and rehabilitation centres. The form consisted of a Microsoft Excel spreadsheet as well as a printable data collection sheet. Information requested for each observation comprised: date, place/address, latitude and longitude in decimal degrees, affected species and numbers (in one column), status (“sick (alive)/dead”), source of information, “Healthy birds of the same species,” “Other species seen,” “other details” and test date and results (if applicable). The template was revised later to separate the columns for species and numbers affected. The completed form was submitted either to a supervisor who collated reports and sent them to veterinary services or reports were submitted directly via email.

Some reports were not submitted in the requested format, and sometimes this resulted in lost data, but often additional data were supplied. This additional data included age, what actions werecarried out with the bird or carcass, whether the observation was made as part of a scheduled patrol, a reference number assigned to the bird, and sex determined on postmortem examination at a rehabilitation centre. A database of laboratory test results was maintained in parallel and cross-referenced with the reports to avoid duplication and eliminate suspected cases that had tested negative.

The number of cases, suspected cases, negative tests from WCPVL, and positive cases from both laboratories are reported for each affected species. Negative tests performed at other laboratories are not available but are believed to be few. Relative case numbers at each location are also assessed. Locations within approximately 20 km of each other were grouped as one area. Where samples from multiple birds were pooled for testing, all birds were counted as negative if the pool tested negative, but only one was counted where the pool tested positive.

Preliminary counts of confirmed cases were published previously [5] with data up until 1 May 2018, but the final numbers are presented here, along with the suspected cases and negative tests.

The challenges and lessons learned during the response to the coastal bird HPAI (H5N8) outbreak are discussed and analysed here. Records of decisions and experiences were gained from email and instant messaging records, minutes of meetings and workshops, and personal discussions.

## 3. Results

### 3.1. Confirmed and Suspected Cases

Between December 2017 and May 2018, an estimated 7,526 birds of 20 species putatively affected by H5N8 HPAI were reported from 31 sites along the western and southern coast of South Africa, in the Western and Eastern Cape provinces (Table 1). The majority of suspected cases were reported as having been found dead (7129), and 199 are assumed to have shown clinical signs of HPAI (because they were reported as “sick,” “euthanised,” “alive,” or exhibiting neurological clinical signs or weakness with cloudy eyes), eleven were only reported as weak, and for 187 birds, no status was recorded.

Approximately 55% of positive cases were reported as having clinical signs of HPAI, and about 30% were found dead. The remainder were simply weak, and two were asymptomatic. These estimates are based on 105/116 positive birds with sufficient information.

Information on the type of sample collected for testing is available for 101/119 positive birds. Fifty-two were diagnosed on a single sample: 29 of these on a brain sample, eleven on pooled tracheal and cloacal swabs, seven on tracheal swabs, four on organ samples, and the remainder on other combinations of pooled swabs. Of the 49 birds tested with more than one sample, a brain sample was tested for all but two birds (total brain samples = 76), and all but one (from a common tern with a positive tracheal and cloacal swab pool) of these brain samples were positive. Another 59 tests were carried out on the 49 birds (seven were tested on three or four samples), of which only ten tests were negative. These comprised four of seven cloacal swabs, two of twelve tracheal swabs and four of 34 cloacal, and tracheal swab pools. One each of the negative tracheal and cloacal swab samples was pooled from the same four already-decomposing gannet carcasses.

Approximately 27 sets of samples, pooled from multiple birds, were tested. Half were from two birds, approximately a quarter from three birds, and the maximum number of birds per pool was six. Three pools were positive, originating from three, four, and five birds, respectively, but only three of these twelve birds were counted.

A distinct, newly-introduced variant caused eight outbreaks in poultry in the northern part of the country during the same period. However, a sacred ibis was the only wild bird recorded to be affected [8], and the variant was not detected in seabirds or in provinces where affected seabirds were reported.

#### 3.1.1. Terns (Family Laridae)

**Figure 1:**
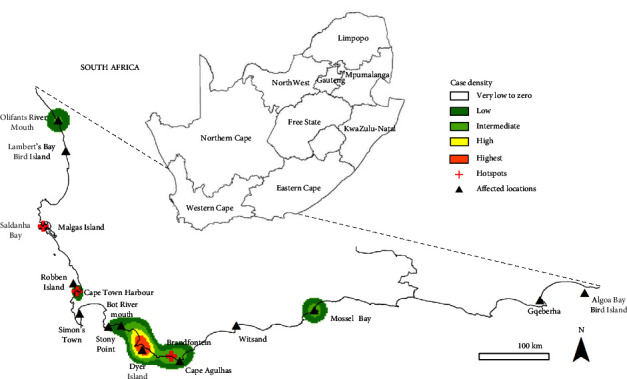
South Africa and its stretch of coastline from which high pathogenicity avian influenza (H5N8) in seabirds was reported between December 2017 and May 2018. The locations with more than 1000 dead seabirds are marked as hotspots. Malgas Island and the roof of a building in Cape Town harbour were the locations of outbreaks at swift tern colonies. The data from these hotspots were excluded from a kernel density estimation (KDE), performed using ArcMap software (v) 10.7.1, ESRI, Redland (USA), with an output cell size of 2 km and a search radius of 30 km. The results of the KDE are shown as a heat map indicating relative case density.

Swift terns (greater crested) (*Thalasseus bergii* Lichtenstein, 1823) accounted for most suspected and confirmed cases (Table 1). 5209 of 5421 swift terns were found dead, 112 were assumed to have shown clinical signs, ten were only weak, and 90 were of unknown status. Most tests performed on the species proved positive, and of the sixteen positive swift terns with age recorded, ten were adults, and six were juveniles. However, severe mortality (>60%) was observed among chicks and juveniles at two colonies on Malgas Island and at Cape Town harbour (Figure 1), with fewer than twenty dead adults observed at the harbour. These events accounted for over 90% of suspected cases, and only seven chicks and 82 juveniles were reported from elsewhere. Seventy-four juveniles were from Dyer Island, mostly in late March.

**Figure 2:**
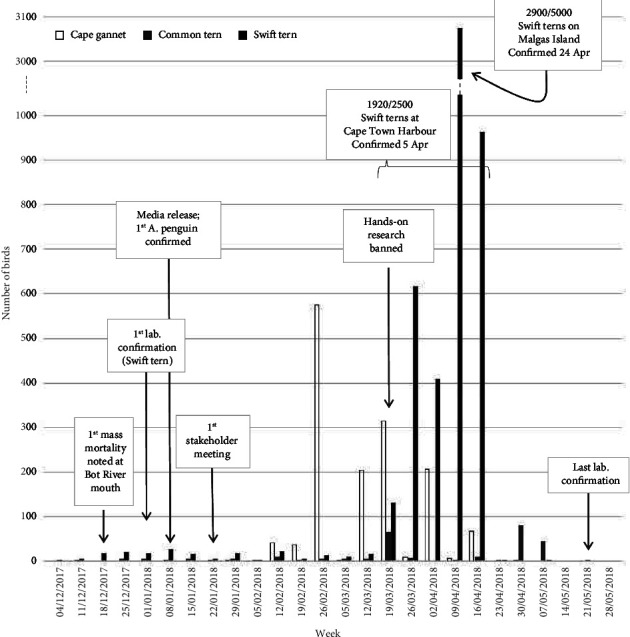
Weekly numbers of dead Swift terns, Common terns, and Cape gannets reported in South Africa during an outbreak of high pathogenicity avian influenza (H5N8) from December 2017 to May 2018. Dates of key observations, laboratory (lab.) confirmations, and response events are indicated by arrows.

**Figure 3:**
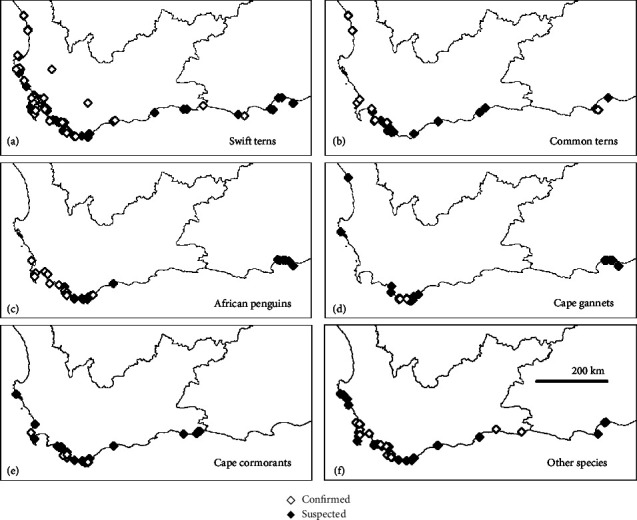
Locations along the South African coastline of different species of seabirds that were suspected and confirmed (via rRT-PCR) to have died from pathogenicity avian influenza (H5N8) between December 2017 and May 2018.

Admissions of sick swift terns to rehabilitation centres increased in early December 2017 (Figure 2). The first noticeable mortality event involving three tern species (mostly swift terns) and two gull species was observed on 18 December at Bot River mouth (Figure 1) [5]; however, the carcasses were too decomposed for AI virus testing. The first positive AI test in a seabird was from a swift tern found on 20 December 2017, followed by regular reports of swift tern carcasses and the high mortalities at the two large colonies affected in March and April. The distribution covered most of the coastline from 200 km north of Cape Town, on the west coast, to Gqeberha (previously Port Elizabeth) in Algoa Bay, approximately 700 km to the east (Figure 3(a)). Clinical signs included initial weakness and inability to fly and “cloudy eyes,” likely due to corneal oedema. Affected birds then developed neurological signs, including head tremors, ataxia and circling, seizures, and death. Two of the positive chicks were apparently healthy, however. Two cases were reported 75 km inland, suggesting severely impaired navigational ability because swift terns usually do not travel more than about 3 km from the coast [21]. Further details are provided in the supporting information.

Deaths in common terns (*Sterna hirundo* Linnaeus, 1758) had a similar temporal and spatial distribution to swift terns (Figure 3) but with lower morbidity and mortality (See supporting information).

#### 3.1.2. African Penguin

Detection of the HPAI virus in endangered African penguins (*Spheniscus demersus* Linnaeus, 1758) caused significant concern, though fewer carcasses were found, and proportionally fewer proved positive than swift terns (Table 1). Ninety-nine of 118 suspected HPAI African penguin cases were found dead. Of the reports based on live birds, fourteen are assumed to have had clinical signs, one only had difficulty breathing, and four were of unknown status. One penguin was found with only a head injury but developed neurological signs and tested H5N8 PCR-positive. Of the 92 suspected penguin cases where age was recorded, 75 were adults, 12 juveniles, and five chicks. Of the 25 confirmed cases with age data, 22 were adults, and three were juveniles. Sex data were available for seventeen positive birds examined postmortem: thirteen were females.

The first infected African penguin was reported on 12 January 2018 (Figure 2), approximately 100 km east of Cape Town and 10 km east of Bot River mouth. Cases were detected between Cape Town and Gqeberha until May 2018 (Figure 3). Fifty dead or moribund penguins were detected in the False Bay area, southeast of Cape Town, including nineteen birds from the Simon's Town colony (five confirmed cases), between 29 January and 30 March and eight from the Stony Point colony (five confirmed) in April and May. Four positive and eleven suspected cases were reported from Dyer Island, further east, between February and May, and three penguins from Robben Island, north of Cape Town, tested PCR-positive in May. Ten suspects and one positive case were reported from Algoa Bay (Gqeberha).

Clinical signs in penguins included mucoid ocular discharge, cloudy eyes, apparent blindness, lethargy and an inability to stand, open-mouthed breathing, and neurological signs such as head or whole-body tremors, head tilt, dorsal neck flexion, bilateral nystagmus, and seizures. A penguin with mild neurological signs was treated supportively and with 35 mg oseltamivir twice a day, under isolation, for 3 weeks. Although a PCR test after two weeks of treatment indicated that virus shedding may have ceased, the bird was euthanized due to a deteriorating neurological state. A postmortem PCR test of the brain was positive for the H5N8 virus. Incidentally, a serum sample taken before euthanasia was also tested for avian influenza antibodies, and results were consistent with what would be expected in poultry (SANCCOB unpublished data, 2022; see supporting information).

#### 3.1.3. Cape Gannet

Approximately 1,500 Cape gannet (*Morus capensis* Lichtenstein, 1823) carcasses were found between February and April 2018 on beaches west of Cape Agulhas, the southernmost tip of Africa (Figures 2 and 3). Age was recorded for half of these birds; 40 were recorded as juveniles, and the remainder were adults. However, there was no corresponding increase in mortality in the three South African breeding colonies. Only two pooled samples tested PCR-positive, while at least another twenty tests were negative (Table 1). Additionally, 60 healthy birds sampled at the Malgas Island breeding colony in February tested negative, and no clinically-affected birds were recorded. Other seabird carcasses were found near Cape Agulhas in much smaller numbers, and carcasses overall were in various stages of decomposition, with signs of substantial scavenging and likely predation, possibly by seals. Smaller numbers of gannet carcasses were found at later surveys in November 2018 and January and March 2019 and were attributed to seal predation.

#### 3.1.4. Cape Cormorant

Most suspected and confirmed cases of Cape cormorants (*Phalacrocorax capensis* Wahlberg, 1855) were reported from the south coast, east of Cape Agulhas (Figure 3). Sixty of 104 Cape cormorants were reportedly found dead. Of the 41 live birds, 24 displayed neurological signs, and these were the source of five out of six confirmed cases (Table 1). A large number (180) of juvenile, emaciated Cape cormorants without neurological signs were found in and around Cape Town and were admitted for rehabilitation in the same period (SANCCOB unpublished data, 2022). However, the number admitted is not unusual, and these were not tested nor counted as suspected cases.

Suspected and confirmed cases in other species, reported in smaller numbers, are described in Table 1 and Figure 3(f).

### 3.2. Outbreak Response

#### 3.2.1. Communication

The national veterinary authorities were first notified of the detection of HPAI in swift terns on 5 January 2018, the day that laboratory confirmation was received by Western Cape provincial veterinary authorities, and twenty days after the first positive samples were taken. The public was informed four days later via a media release (Figure 2) [22]. A contact list of stakeholders was developed as a priority to provide information and advice on outbreak management.

Western Cape provincial veterinary services provided general advice on outbreak response and reporting, while rehabilitation centres issued more specific guidelines related to their own functioning, including admission of sick and dead birds. Summaries of information with specific guidelines were distributed among conservation staff members [23]. The first meeting with stakeholders was held in mid-February after the virus was detected in penguins (Figure 2). Monthly meetings occurred between the leading role-players (Government veterinary and environmental agencies and conservation authorities) to adapt response activities and report progress.

Regular media statements were released to keep the public informed and to advise on handling dead and sick birds. These prompted queries from journalists, especially after the news that infection was detected at the mainland penguin colony in Simon's Town, near Cape Town, a major tourist attraction [24].

#### 3.2.2. Outbreak Monitoring and Reporting

The initial media release requested the public to report suspected cases to their local state veterinary or government office. An official request for two-weekly reports, citing relevant legislation, was issued in early March to government veterinary offices, relevant conservation authorities, and rehabilitation facilities. The standardized template for reporting observations was supplied.

Four hundred and twenty-seven (427) reports (each related to a single site and date) were received by Western Cape Veterinary Services: approximately 55% from rehabilitation centres, mostly from reports by the public, 20% from the provincial conservation authority (Cape Nature), 11% from South African National Parks (but 40% of suspected cases), and the rest from other sources such as BirdLife South Africa, the public, municipal staff, private vets, Cape Town aquarium staff (reporting 25% of suspected cases), and the SPCA. Forty-nine reports were excluded because they related to birds that later tested negative (4), with unspecified numbers (“lots,” “several”), where species was not determined (e.g., “Tern”: 27 reports, 169 birds, “Cormorant;” (2), no species (2)) or related to species with only single reports or a small number of birds found dead, and with no laboratory confirmation in the species (14 reports). Of 40 reports from the provincial authority, twelve were from scheduled patrols, and 28 were “ad hoc observations.” Very few reports included information on healthy birds observed near suspected cases.

Data were received from rehabilitation centres and conservation authorities after formatting and cleaning, so they were generally of good quality and easily collated by that stage. Some problems encountered included missing geographic coordinates, or no location stated at all, or location names without coordinates that could not be traced. Another challenge was duplication of reports when the conservation authority sent birds to a rehabilitation centre, and both organisations reported them. This was particularly difficult to resolve if different dates were recorded, e.g., the date found and the date of arrival at the rehabilitation centre.

The data received were first published as an interactive Google map, accessible online to anyone with the web address, at the end of March and then as a status update in May, amended in September.

#### 3.2.3. Response in the Wild

The goals of managing the disease in the wild were to minimize viral spread and human-induced stress to wild populations. Actions were limited to removing carcasses and sick birds as sources of the virus, where possible, and limiting the additional mechanical spread of the virus and disturbance caused by human activities.

Activity in the colonies was minimized by banning all hands-on research activities in late March [25, 26] and restricting monitoring activities to remote methods (Figure 2). In June, after the first penguin breeding peak and a month since the last confirmed case in a coastal bird, it was decided that low-impact, noninvasive research activities were allowed, with biosecurity protocols to be followed. The protocol provided basic guidelines for the use of disinfectants, including the principles of the correct active ingredient, concentration and contact time, and the negative effects of excessive biological material. Recommendations included waterproof clothing or a plastic apron, rubber boots, and gloves. The waterproof clothing proved extremely uncomfortable in hot weather. In September, more intrusive procedures and guano sampling were permitted to proceed. The insertion of microchips, used to monitor individual birds, was only allowed again in early 2019.

The public was requested via media releases to avoid handling dead bird carcasses, especially if they had contact with domestic birds. At the seabird colonies, conservation authorities distributed information to staff; on the disease and with instructions for biosecurity and managing sick birds and carcasses and for record-keeping. Protective clothing, including gloves as a minimum, rubber boots, disposable aprons, and face masks for added protection, was to be worn in the colonies, especially when handing sick birds and carcasses. Disinfectant was distributed for application to equipment, clothing, footwear, and vehicles. Compliance was high among the staff of management authorities, especially at mainland colonies, and at rehabilitation centres, possibly due to some fear of personal infection. At the two mainland penguin colonies, additional measures were required to manage visitors, including footbaths and restricting access to the raised boardwalks. Notices were also displayed to inform guests of the situation and potential risk to domestic birds. Over the peak outbreak period, the release of African penguins and other seabirds from rehabilitation centres was discontinued at the Simon's Town African penguin colony.

Information about avian influenza in humans was distributed to conservation authorities' staff, with instructions to visit the nearest clinic if flu-like symptoms were experienced. However, it was emphasised that there was no evidence that the HPAI (H5N8) virus had caused any mammal or human infections. The Department of Health was informed of the locations of the important bird sites, and they distributed instructions and sampling equipment to the closest clinics by early April.

Carcass disposal was attempted where possible or necessary in public areas. Guidelines issued by provincial veterinary services in April listed disposal options and provided contact details for waste management and air quality control officials and carcass disposal experts. It was suggested that carcass masses of less than 10 kg be buried at least 1 m deep with lime above the high tide mark. This was given special approval by provincial waste management authorities since the routine burying of infected carcasses is illegal under national waste management legislation. The swift terns from Cape Town harbour were incinerated, which accounts for approximately 25% of carcasses. Approximately 40% from Malgas Island were either buried on the island or burnt in pyres. Another 13% were buried along the coast, in the sand above the high water line, close to where they were found. The disposal method is unknown for 18% of suspected cases reported. Some would have been submitted to a rehabilitation centre and, therefore, would have been incinerated. These were transported in a triple layer of plastic bags. It is expected that some were also left on the beach. Those carcasses that were removed were collected by hand, by staff wearing protective clothing that included gloves, overalls, and boots, and masks when carcasses were burnt. A disinfectant footbath and spray were also used at the harbour.

Euthanasia was advised for swift terns with suspicious clinical signs, given that treatment had already proven unsuccessful. Private veterinarians, without frequent bird patients, assisted with this. Penguins with mild or moderate signs were accepted for assessment at the rehabilitation centres, given their endangered status.

#### 3.2.4. Management in Seabird Rehabilitation Centres

The provincial veterinary authorities considered quarantine of seabird rehabilitation centres in the Western Cape but did not implement it. Preventing admission of new patients to rehabilitation centres was deemed unreasonable, given their function. Though prerelease testing was carried out at the largest seabird centre between mid-February and the end of March, no positive results were obtained, and it was discontinued. Increased biosecurity measures were instituted to protect the rehabilitation centres. These included the use of footbaths, improved disinfection, isolation facilities for suspected AI cases, and the euthanasia of severely affected cases without admission to the rehabilitation centre. The two main seabird centres were advised mainly by in-house or contracted veterinarians. One shared its information sheet and protocol with smaller centres handling a wider range of species, conservation authorities, and veterinary practices. This information covered the collection of suspected cases, transport to and handling at the centre, equipment needed, isolation procedures, waste disposal, reporting requirements, and sample collection.

Available records from the largest seabird rehabilitation centre reflect that birds euthanised specifically on suspicion of HPAI, from January to May, included 51 swift terns, of which twelve were tested, and one was negative, ten common terns (one was tested and positive), five penguins (one tested negative: a chick), and two Cape cormorants (one tested negative, the other was not tested). These data may not be completely accurate because record keeping was difficult, and many more already-dead birds were handled, but stricter record-keeping protocols were instituted as the outbreak progressed. Treatment was attempted in a few penguin cases, but they usually died within 12 hours of admission or were euthanized to prevent further suffering. In comparison, in the same period, the centre released 165 African penguins, 154 gulls, 34 Cape cormorants, one Cape gannet, eight birds of other species, and zero swift or common terns.

## 4. Discussion

African penguins, Cape gannets, and Cape cormorants are classified as endangered on the IUCN Red List and were all impacted by the South African HPAI (H5N8) epidemic in the austral summer and autumn of 2018 [5]. There was considerable concern that the disease could hasten their population decline, especially because the successful management of wildlife disease is challenging, and options are particularly few for HPAI. A better understanding of the disease's epidemiology and systematic assessment of any available control measures are needed to develop effective response protocols.

The last recorded outbreak of HPAI in seabirds in South Africa, and the first isolation of AIV from wild birds worldwide, occurred in April and May 1961. Mainly common terns were affected, with at least 1300 dying in an area similar to that affected in 2018 [27, 28]. The observed higher mortality of swift terns in 2018 could therefore be considered surprising. Common terns, as migrant summer visitors, are also present in higher numbers than swift terns at that time of year, although swift terns are the only resident species of tern that breed in such large numbers on the west and south coast of southern Africa [21, 29]. Some carcasses may have been misidentified by inexperienced observers, who had heard that the disease had initially been detected in swift terns, but the 60% chick mortality in two of the four main swift tern colonies (Department of Forestry, Fisheries and the Environment (DFFE) unpublished census data, 2022) is in excess of the normal 20% first year mortality estimated by Payo-Payo et al. [30]. Although most swift terns were found dead, without clinical signs having been observed, and relatively few tests were carried out, the high proportion of positive tests in terns and above-normal mortality rate suggests that the majority died from HPAI.

Census data from DFFE show that the two worst-affected swift tern colonies were, in fact, much larger in 2018 (at least twice and four times as large, respectively) than in previous years. In contrast, sites that usually have the largest colonies had 60–80% of the previous year's number of breeding pairs, while no breeding was recorded at other, usually smaller, colonies. It is, therefore, possible that high colony density contributed to the high mortality rates at the two affected sites. No breeding was recorded at these sites in 2019. The census data also indicates a decrease in the total number of swift tern breeding pairs since 2018; in 2019, it was approximately half that of 2018. However, by 2021 the population appeared to have recovered somewhat and was similar to relatively low numbers recorded in 2012/2013. The biology of the species is such that recovery within three years is highly unlikely, so it may be that the 2019 census led to an underestimation of numbers.

Fortunately, the African penguin mortality rate did not accelerate, even after they congregated for breeding, and no chicks tested positive. There was also no obvious subsequent change in the trend of the population or colony breeding pair numbers. Nest densities are highest in Simon's Town and at Stony Point and Dyer Island, where most cases were recorded, and this could have played a role, but the final number of suspected cases was relatively low, given a population size similar to swift terns [29]. Additionally, given the high proportion of negative tests, the suspected cases could be an overestimation of affected individuals. As a charismatic and easily-identified species, there may have been more energetic reporting of sick and dead African penguins. However, Namibia's outbreak, almost a year later, is believed to have killed at least 450 penguins between January and April 2019 and, in this outbreak, African penguins seem to have been the only species affected [31, 32], so they are not necessarily more resistant to HPAI than terns.

Investigating Cape cormorant and Cape gannet deaths was challenging because there were no clear indication that the suspected increased mortality rates were due only to HPAI, and no definite conclusion on the cause of death has been reached. Fresh samples from gannets were scarce, and budget constraints limited samples in cormorants to birds with suspicious symptoms. The outbreak coincided with the season when young cormorants fledge and are found around Cape Town in an emaciated state (SANCCOB, unpublished data). However, such high numbers of gannet carcasses are not common in southern Africa, especially away from the colonies. Avian cholera (caused by the bacterium *Pasteurella multocida*) has caused deaths in colonies, and thousands of fledglings have been killed by Cape fur seals (*Arctocephalus pusillus* Schreber 1775) around a colony [33], but this level of mortality away from the colonies is unusual. The DFFE census data do not indicate any obvious change in population trends in either of these species since 2018.

A more systematic approach to sampling, aiming for better representativeness, could have helped both to estimate species susceptibility with more confidence, and to obtain the best possible sample set of circulating viruses, to perform genetic sequencing. However, careful planning, communication, and budgeting are required to achieve this. Samples must be collected and packaged correctly by trained staff and transported to an approved laboratory with sufficient testing capacity. Fresh samples and/or the use of a protein-rich viral transport medium containing antibiotics for swabs are vital to provide virus for genetic sequencing to help elucidate virus transmission pathways. A formal surveillance and testing program, with required resource estimates, should be developed as part of contingency plans. Sufficient, representative samples should be obtained from both clinically-affected birds and apparently healthy individuals of both affected and possible subclinical carrier species. However, the associated risk of mechanical disease spread and colony disturbance must also be considered.

Earlier detection of the disease could have allowed more time for improved monitoring and preparation. However, funding was limited, and the seabird outbreak was unexpected so many months after the outbreaks in domestic species. The original expectation was also that it would manifest as a respiratory rather than a neurological disease, although research on cases in water birds in Europe may have dispelled this misconception [34].

Monitoring of the outbreak was performed via a rapidly developed, ad hoc system that was adapted based on the information that was produced. There was a lag before the monitoring began while instructions were distributed, and it was impossible to collect sufficiently detailed data or samples to establish the primary source or means of transmission. Baseline mortality and morbidity data were also lacking, which added to uncertainty about the actual effect of the disease. It was only possible to estimate relative mortality rates and susceptibility in different species and estimate the spatial distribution, and try to gauge when more intervention could become necessary, especially in colonies. This basic information should be a minimum requirement in an outbreak investigation. The aim was to prioritise the essential data and try to ensure that these were recorded efficiently. Insistence on precise counts was necessary as hysterical and exaggerated descriptions were common. However, it was demonstrated that additional epidemiological information such as age, signs of disease, or cause of death and action taken could be recorded under most circumstances. This is likely to be more difficult when there are many carcasses or sick birds to manage in a short time and if a larger number of, likely unskilled, personnel are needed, but basic training and perhaps an assigned record-keeper could make this possible.

Reporting was possibly biased to areas inhabited or visited by people. However, data collected should aid decision-making and be focused on vulnerable species. The intensified monitoring in penguin colonies may therefore have been sufficient without any additional patrols on more remote stretches of coastline. A central reporting point, which could then provide feedback, was a great advantage, and the template facilitated the rapid amalgamation of standardized information from different sources. However, a digital system, such as a mobile phone or web-based application, would have made it simpler to transmit accurate records, including automatically collected date and location, to a central point and allow the provision of photographs to aid species identification. Additionally, systematic surveillance, utilizing a network of observers [35] reporting at regular intervals from known locations, would have significantly improved the quality of the data. It could have provided a better picture of surveillance efforts to establish which areas were truly unaffected and which were simply neglected. Gathering baseline mortality data outside of outbreak periods should also be a priority. This will ensure early detection of increased mortality rates and that the correct data are collected from the beginning of any outbreak to allow a better understanding of the epidemiology of the disease.

Brain swabs and a pooled tracheal and cloacal sample have been used as diagnostic samples at the WCPVL since the HPAI outbreaks in poultry in 2017. The use of the brain sample was based on both the observed neurological clinical signs, indicating central nervous system involvement, and the need for easily accessible tissue samples that would cause the least contamination of the work area. The sample can be taken quite cleanly, and it saves opening the whole carcass, especially where a full postmortem examination is not possible or desired, and the only aim is to determine the presence or absence of the HPAI virus. Additionally, the brain is protected by the skull and may be better protected from desiccation in older carcasses. Conclusions that can be drawn from these data are limited by the scarcity of other organ samples. However, the high proportion of birds with both positive brain samples and tracheal and (or) cloacal samples indicates that where birds were determined to be shedding the virus via the respiratory and (or) digestive tracts, and the virus was also present in the brain. This is aligned with findings by Swayne [36], who found high virus (HPAI H5N1) replication titres in the brains and hearts of a variety of bird species, and Van Den Brand et al. [37], who found the H5N1 virus most consistently at the highest concentrations in the brains of raptors and with Caliendo et al. [38] who recorded a high level of virus neurotropism in Eurasian wigeons and barnacle geese. There is also an indication from data presented here that tracheal and (or) cloacal samples, without organ samples, may not be reliable in detecting the HPAI virus in seabirds. To understand the pathogenesis and epidemiology of the HPAI in seabirds, it is necessary to conduct more detailed postmortem examinations to determine sex, reproductive stage, and other possible risk factors and to sample, test, and study more individual organs. However, this requires planning in terms of infrastructure, biosecurity, expert personnel, and funding.

Effective communication with relevant stakeholders was essential and should be included in all contingency plans. It is both necessary to collect all available data about cases and affected locations and also to give feedback on the overall progression of the outbreak and on management decisions. Communication about the outbreak was relatively straightforward once a list of stakeholders had been assembled but was hampered before that had been achieved. The list also allowed redirecting of questions from journalists to the correct organizations or people. When compiling media statements, providing additional information to public relations personnel (lists of frequently asked questions) was efficient in assisting with follow-up questions from journalists.

There was little experience with and preparation for the South African HPAI (H5N8) outbreaks in coastal birds in 2018. Broad guidelines for disease response had been compiled for the African Penguin Biodiversity Management Plan [39] but had not been implemented at colonies. This resulted in a high level of uncertainty and lack of confidence when making decisions about outbreak response and with limited management options. Veterinary Services had gained some experience dealing with the virus during the 2017 HPAI outbreaks in poultry, but under different, more controlled conditions.

Minimising colony disturbance was prioritised, so although carcass removal was perceived as ideal, it was ultimately not considered essential, especially under the dry climatic conditions experienced at the time. HPAI viruses are expected to be present in organs and muscles of dead birds and can be transmitted by ingestion of these tissues, but this may require a higher virus dose than via the respiratory route [40]. Scavenging species may therefore be at risk. In this outbreak, scavengers such as kelp gulls appeared resistant, but the infected jackal buzzard reported here, and pied crows (*Corvus* albus Statius Müller 1776) diagnosed in 2018 [5] may represent larger numbers of affected scavenging birds that would have been more difficult to detect than colonial species. Scavengers may also cause the spread of the virus to other species, perhaps creating aerosols or droplets while feeding or playing a role in mechanical transmission, possibly by contaminating water sources. However, further research is required to determine whether an undisturbed infected carcass could be a source of virus for nonscavenging species and, if so, under what environmental conditions.

It was not possible to provide blanket guidelines for carcass disposal, as the conditions differ between locations, but better planning could have ensured that the best option was chosen. Suitable ground for burial was often lacking, especially on the island seabird colonies. Space and plant material were also often unavailable for composting, and transporting carcasses to approved landfill sites was not encouraged to prevent the spread of the virus. Additionally, incineration facilities were few and costly but were used for smaller volumes of carcasses. Staff to collect carcasses were not always available, but concerns were also expressed that regular carcass collection may also cause harmful disruption to nesting seabirds within the colonies.

The decision to allow the mainland seabird colonies to remain open to tourists was based on the expectation that, with the already widespread distribution of the disease and the high mobility of the birds, visitors were unlikely to contribute significantly to virus spread. Raised walkways limit contact of visitors with potentially infectious material, and barring visitors would have cut off an important revenue stream necessary to maintain and protect the colonies. The ban on research was possibly too drastic in hindsight and disrupted some valuable long-term projects, but minimizing direct human contact with the birds seemed reasonable at the time. The spread of the virus between birds was considered a more important mode of transmission than human-assisted transmission, but human disturbance and handling were considered a source of stress that could result in immunosuppression.

Rehabilitation centres had to be managed differently from poultry farms, though the inclination initially may have been to resort to principles applied to farms, such as quarantine and culling. The cost of prerelease testing was prohibitive, and the risk posed by releasing a healthy carrier was considered negligible, given the assumed high levels of virus in the environment. The threat posed by virus introduction to the centres was also a sufficient incentive for the centres to take all possible precautions without being under official quarantine. They appear to have been successful, as no birds developed signs of avian influenza while in the facilities. However, determining which patients could be safely admitted to a rehabilitation centre during the outbreak was challenging without sensitive rapid point-of-care tests for AIV, which are currently not allowed at all in South Africa.

Contingency plans for future outbreaks should include ongoing training of relevant personnel in good biosecurity, data collection, identification and management of affected birds, and other possible appropriate interventions. Basic biosecurity should be maintained, even between outbreaks, as the disease may go unrecognised in the early stages. Stocks of sampling equipment and personal protective equipment should be maintained, and carcass management plans should be in place. Centrally, there needs to be clear communication about different organizational and personnel roles. Ideally, there should be one coordinator per province dedicated to managing the outbreak and receiving regular progress reports. This may also be the best person to coordinate media statements. Resource requirements should be an explicit part of contingency planning, and there needs to be clarity on how funding will be accessed and distributed.

An understanding of the epidemiology of HPAI in seabirds is still lacking, however, and prevents the formulation of truly effective contingency plans. The importance of carcass removal is still undetermined, and the study of virus survival in and transmission from carcasses requires research. A review of current data on virus survival under coastal environmental conditions and the identification and filling of data gaps are also needed. A better understanding of the role of subclinical carrier species and transmission routes (e.g., respiratory vs. alimentary tract) could also shed light on other possible interventions, such as managing virus load in the environment. Data on baseline mortality would ensure that response is appropriate and prompt if required. Vaccination, as a method of preventing infection, virus shedding and (or) disease, appears impractical for wild populations at this stage. However, available vaccines and vaccine technology should be explored to assess possible situations where vaccination may become feasible.

Given limited response options and an understanding of HPAI epidemiology in seabirds, it is vital that all future observations and lessons learned continue to be recorded and discussed.

## Figures and Tables

**Table 1:** Confirmed and suspected cases of high pathogenicity avian influenza (H5N8) in wild birds in Western and Eastern Cape provinces, South Africa (December 2017 to May 2018).

Species	Date 1st positive case found	No. suspected/positive cases reported	No. birds tested positive (rRT-PCR)	No. birds tested negative at WC PVL^†^	No. positive areas
African oystercatcher (*Haematopus moquini* Bonaparte, 1856)	09 Mar 2018	5	1	2	1
African penguin (*Spheniscus demersus* Linnaeus, 1758)	12 Jan 2018	118	31	17	8
Arctic tern (*Sterna paradisaea* Pontoppidan, 1763)	19 Dec 2017	10	0	0	0
Cape cormorant (*Phalacrocorax capensis* Wahlberg, 1855)	15 Feb 2018	104	5	15	3
Cape gannet (*Morus capensis* Lichtenstein, 1823)	15 Feb 2018	1473	2	20	1
Columbidae (Doves/Pigeons)	18 Jan 2018^‡^	Not recorded	5	45	3
Common tern (*Sterna hirundo* Linnaeus, 1758)	04 Jan 2018	288	8	1	8
Crowned cormorant (*Microcarbo coronatus* Sparrman, 1788)	02 Mar 2018	2	1	0	1
Egyptian goose (*Alopochen aegypticus* Linnaeus, 1766)	16 Jan 2018	2	1	2	1
Grey-headed gull (*Larus cirrocephalus Vieillot,* 1818)	23 Feb 2018	1	1	0	1
Hartlaub's gull (*Chroicocephalus hartlaubii* Bruch, 1853)	15 Feb 2018	24	8	21	4
Jackal buzzard (*Buteo rufofuscus* JR Forster, 1798)	11 Jan 2018	1	1	0	1
Kelp (Cape) gull (*Larus dominicanus* Lichtenstein, 1823)	15 Nov 2017	17	0	16	0
Parasitic jaeger (*Stercorarius parasiticus* Linnaeus 1758)	02 Apr 2018	1	1	0	1
Sabine's gull (*Xema sabini* Sabine, 1819)	25 Feb 2018	1	0	1	0
Sacred ibis (*Threskiornis aethiopicus* Latham, 1790)	30 Mar 2018	18	0	11	0
Sandwich tern (*Thalasseus sandvicensis* Latham, 1787)	27 Jan 2018	23	2	1	2
Spotted eagle-owl (*Bubo africanus* Temminck, 1821)	22 Feb 2018	1	1	0	1
Swift tern (*Thalasseus bergii* Lichtenstein, 1823)	20 Dec 2017	5421	46	4	23
White-breasted cormorant (*Phalacrocorax lucidus* Lichtenstein, 1823)	21 Feb 2018	16	0	0	0
Total		7526	115	83	31

^†^Western Cape Provincial Veterinary Laboratory rRT-PCR testing 1 December 2017 to 5 September 2018. The number of total tests carried out on wild birds at other laboratories is not available. ^‡^Only cases between December 2017 and May 2018 are counted here, but the first detection was in August 2017.

## Data Availability

The suspected and confirmed case data used to support the findings of this study may be released upon application to Laura Roberts, who can be contacted at Laura.Roberts@westerncape.gov.za.

## References

[1] Stallknecht D. E., Brown J. D., Swayne D. E. (2017). Wild bird infections and the ecology of avian influenza viruses. *Animal Influenza*.

[2] Sims L., Harder T., Brown I. (2017). Highly pathogenic H5 avian influenza in 2016 and 2017 – observations and future perspectives. *EMPRES Focus On*.

[3] Ndumu D., Zecchin B., Fusaro A. (2018). Highly pathogenic avian influenza H5N8 clade 2.3.4.4B virus in Uganda, 2017. *Infection, Genetics and Evolution*.

[4] Food and Agriculture Organisation of the United Nations (FAO) (2017). *H5N8 HPAI in Uganda: Further Spread in Uganda and Neibouring Countries*.

[5] Khomenko S., Abolnik C., Roberts L., Waller L., Monne I., Shaw K. (2018). 2016–2018 spread of H5N8 highly pathogenic avian influenza (HPAI) in sub-saharan Africa: epidemiological and ecological observations. *EMPRES Focus On*.

[6] Abolnik C., Pieterse R., Peyrot B. M. (2018). The incursion and spread of highly pathogenic avian influenza H5N8 clade 2.3.4.4 within South Africa. *Avian Diseases*.

[7] Fusaro A., Zecchin B., Vrancken B. (2019). Disentangling the role of Africa in the global spread of H5 highly pathogenic avian influenza. *Nature Communications*.

[8] Abolnik C. (2019). Outbreaks of clade 2.3.4.4 H5N8 highly pathogenic avian influenza in 2018 in the northern regions of South Africa were unrelated to those of 2017. *Transboundary and Emerging Diseases*.

[9] Peyrot B. M., Abolnik C., Anthony T., Roberts L. C. (2022). Evolutionary dynamics of the clade 2.3.4.4B H5N8 high pathogenicity avian influenza outbreaks in coastal seabirds and other species in southern Africa from 2017-2019. *Transboundary and Emerging Diseases*.

[10] European Food Safety Authority (EFSA) (2022). Avian influenza overview March – June 2022. *EFSA Journal*.

[11] European Food Safety Authority (EFSA) (2022). Avian influenza overview December 2021 – March 2022. *EFSA Journal*.

[12] Tey A. (2022). Avian flu threatens seabird nesting colonies on both sides of the atlantic. https://www.audubon.org/news/avian-flu-threatens-seabird-nesting-colonies-both-sides-atlantic.

[13] Ramey A. M., Hill N. J., Deliberto T. J. (2022). Highly pathogenic avian influenza is an emerging disease threat to wild birds in north America. *Journal of Wildlife Management*.

[14] Peterson M. J., Ferro P. J., Silvu N. (2016). Wildlife Health and disease: surveillance, investigation, and management. *The Wildlife Techniques Manual*.

[15] Cromie R. L., Lee R., Delahay R. J. (2012). Ramsar wetland disease manual: guidelines for assessment, monitoring and management of animal disease in wetlands.

[16] Kleyheeg E., Slaterus R., Bodewes R. (2017). Deaths among wild birds during highly pathogenic avian influenza A(H5N8) virus outbreak, The Netherlands. *Emerging Infectious Diseases*.

[17] Slomka M. J., Pavlidis T., Banks J. (2007). Validated H5 eurasian real-time reverse transcriptase-polymerase chain reaction and its application in H5N1 outbreaks in 2005-2006. *Avian Diseases*.

[18] European Union Reference Laboratory for Avian Influenza and Newcastle Disease (2021). SOP VIR 143 Detection of Eurasian H5 Avian Influenza Virus by Real-Time RT-PCR. https://www.izsvenezie.com/documents/reference-laboratories/avian-influenza/diagnostic-protocols/sop-vir-143.pdf.

[19] Hoffmann B., Hoffmann D., Henritzi D., Beer M., Harder T. C. (2016). Riems influenza a typing array (RITA): an RT-qPCR-based low density array for subtyping avian and mammalian influenza a viruses. *Scientific Reports*.

[20] European Union Reference Laboratory for Avian Influenza and Newcastle Disease (2021). SOP VIR 1004 HA and NA Subtyping of Avian Influenza Virus by Real-Time RT-PCR. https://www.izsvenezie.com/documents/reference-laboratories/avian-influenza/diagnostic-protocols/sop-vir-1004.pdf.

[21] Hockey P. A. R., Dean W. R. J., Ryan P. G. (2005). Roberts Birds of Southern Africa. *Trustees of the John Voelcker Bird Book Fund*.

[22] Western Cape Government (2018). Avian influenza update: spread amongst poultry has halted. https://www.westerncape.gov.za/news/avian-influenza-update-spread-amongst-poultry-has-halted.

[23] Kock A. (2018). *Highly Pathogenic Avian Influenza (HPAI H5N8) Cases in SANParks: Seabirds January – April 2018*.

[24] South African National Parks (SANParks) (2018). Avian (bird flu) outbreak confirmed at Boulders penguin colony. https://www.sanparks.org/about/news/?id=57449.

[25] Department of Environmental Affairs (2018). Avian influenza outbreak on seabirds. https://www.environment.gov.za/mediarelease/avianinfluenzaoutbreakonseabirds.

[26] Wild S. (Nature 2018). Avian Flu Freezes Coastal Bird Research in South Africa. https://www.nature.com/articles/d41586-018-03951-6.

[27] Rowan M. K. (1962). Mass mortality amongst European common terns in South Africa in April-May 1961. *British Birds*.

[28] Alexander D. J. (2000). A review of avian influenza in different bird species. *Veterinary Microbiology*.

[29] Ryan P. (2017). *Guide to Seabirds of Southern Africa*.

[30] Payo-Payo A., Sanz-Aguilar A., Gaglio D. (2018). Survival estimates for the greater crested tern Thalasseus bergii in southern Africa. *African Journal of Marine Science*.

[31] Umberto M., Aikukutu G., Roux J. (2020). Avian influenza H5N8 outbreak in African penguins (Spheniscus demersus), Namibia, 2019. *Journal of Wildlife Diseases*.

[32] Cannon J. C. (2019). *Bird Flu in Namibia’s Penguins Wanes, after Killing Nearly 500*.

[33] Sherley R. B., Crawford R. J., Dyer B. M. (2019). The status and conservation of the Cape Gannet Morus capensis. *Ostrich*.

[34] Napp S., Majó N., Sánchez-Gónzalez R., Vergara-Alert J. (2018). Emergence and spread of highly pathogenic avian influenza A(H5N8) in Europe in 2016-2017. *Transbound. Emerg. Dis*.

[35] Lawson B., Petrovan S. O., Cunningham A. A. (2015). Citizen science and wildlife disease surveillance. *EcoHealth*.

[36] Swayne D. E. (2007). Understanding the complex pathobiology of high pathogenicity avian influenza viruses in birds. *Avian Diseases*.

[37] Van Den Brand J. M., Krone O., Wolf P. U. (2015). Host-specific exposure and fatal neurologic disease in wild raptors from highly pathogenic avian influenza virus H5N1 during the 2006 outbreak in Germany. *Veterinary Research*.

[38] Caliendo V., Leijten L., Van De Bildt M. (2022). Tropism of highly pathogenic avian influenza H5 viruses from the 2020/2021 epizootic in wild ducks and geese. *Viruses*.

[39] Parsons N. J., Vanstreels R. E. T. (2016). *Southern African Seabird Colony Disease Risk Assessment*.

[40] Swayne D. E., Beck J. R. (2005). Experimental study to determine if low-pathogenicity and high-pathogenicity avian influenza viruses can be present in chicken breast and thigh meat following intranasal virus inoculation. *Avian Diseases*.

