# Supplementary material for: Descriptive Epidemiology of and Response to the High Pathogenicity Avian Influenza (H5N8) Epidemic in South African Coastal Seabirds, 2018
Source: Transbound Emerg Dis. 2023 Feb 23;2023:2708458. doi: 10.1155/2023/2708458 (PMC12016864; doi:10.1155/2023/2708458)
Supplement: Supplementary Materials — Additional information is provided on suspected and confirmed HPAI cases in terns, African penguins, and other species. [file 2708458.f1.docx]

**Supplemental information**

**Highly pathogenic avian influenza (H5N8) in different species of seabirds in South Africa in 2018**

*Terns*

The first relatively large mortality event was observed at the Bot River mouth on 18 December 2017. Carcasses of thirteen Swift Terns, seven Arctic Terns and one each of a Kelp Gull, Hartlaub’s Gull and Sandwich tern (*Thalasseus sandvicensis*) were counted.

From mid-March to mid-April 2018, the virus was detected at two Swift Tern colonies, on the roof of a building at the harbour in Cape Town (68% mortality among 2500 juveniles) and on Malgas Island, 100 km north of Cape Town (60% mortality among an estimated 5000 chicks). Adult mortality appeared far lower, with 150 dead adults counted on Malgas Island and only 20 from the harbour. By contrast, lower mortality was reported on Dyer Island (100 km south east of Cape Town; 167 birds out of 4000 breeding pairs, 5% mortality), but 100 carcasses were adults. On Robben Island, only one sick bird and one carcass tested positive in May 2018 out of approximately 2000 breeding pairs, and no increase in mortality was reported.

The largest single incident with Common Terns was 62 birds found dead in Mossel Bay, 330 km east of Cape Town in March. However, a larger die off (132 birds) 100 km north of Cape Town (Olifants River Estuary) between mid-April and mid-May 2018 was identified as Common Terns from photographs.

Of interest was a dead sandwich tern, positive on PCR for H5N8 virus, found in Cape Town in January 2018, which was ringed as a chick at Terschelling Oosterend, Friesland, Netherlands (SAFRING pers. comm).

*African Penguin*

The serological tests performed on the penguin involved in attempted treatment, on samples taken before euthanasia, were done at the Western Cape Provincial Veterinary Laboratory. First, an AI multiscreen enzyme-linked immunosorbent assay (ELISA) (IDEXX AI MultiS-Screen Ab Test (99-12119) was performed, directed against antibodies against the nucleoprotein and avian influenza antibodies were detected. Next, the serum was analysed with haemagglutinin inhibition (HI) tests using a clade 2.3.4.4 H5N8 antigen, a H5N1, H5N2, H6N8, H6N2, H7N1 and H7N7 antigens. Positive titres (≥1:16) were detected for all H5 antigens (highest on H5N8) and H6N8, as is expected for antibodies to an H5N8 infection.

*Other species*

Gull carcasses were reported regularly starting in January 2018, but not in large numbers. One Hartlaub’s Gull (of six tested) and one Grey-Headed Gull tested positive in February 2018. However, five of six Hartlaub’s gulls found around Cape Town, tested positive in April, and another positive was found at Blouberg beach on 11 May. There was a report of a Hartlaub’s Gull on Robben Island in the middle of July that looked suspicious, but a cause could not be confirmed. A few individual Kelp Gulls and some from a batch of fourteen dead and dying on a beach, with eleven dead Hartlaub’s Gulls, near Saldanha Bay in early March 2018 tested negative. One Kelp Gull recovered after treatment with activated charcoal.

Concurrently with the avian influenza outbreaks, in both poultry and wild sea birds, large numbers of dead pigeons and doves were reported across the Western Cape. Ten individuals (10%) were positive for avian influenza, but half of the birds tested were PCR-positive for avian paramyxovirus virus type 1 (that can cause Newcastle disease), confirmed to be a strain called pigeon paramyxovirus (PPMV). Eight birds had a mixed infection with avian influenza and PPMV.
